# The Extracellular Matrix in Liver Regeneration: Biological and Therapeutic Insights

**DOI:** 10.3390/bioengineering13030335

**Published:** 2026-03-13

**Authors:** Haodong Ma, Wenyue Wu, Wen Zhang, Hong Li, Ziyan Pan, Caihong Wang, Ruoyu Gao, Qiushuang Ji, Zhi Chen, Hong You, Wei Chen

**Affiliations:** 1 Beijing Clinical Research Institute, Beijing Friendship Hospital, Capital Medical University, Beijing 100050, China; leslie9866@163.com (H.M.); lihong199612@163.com (H.L.); panziyan0902@163.com (Z.P.); gry10086yrg@163.com (R.G.); 19558557036@163.com (Q.J.); 13479228852@163.com (Z.C.); 2 State Key Lab of Digestive Health, Beijing Friendship Hospital, Capital Medical University, Beijing 100050, China; canacewu@126.com (W.W.); zhangwen0315@163.com (W.Z.); wch2435@163.com (C.W.); 3 National Clinical Research Center of Digestive Diseases, Beijing Friendship Hospital, Capital Medical University, Beijing 100050, China; 4 Liver Research Center, Beijing Friendship Hospital, Capital Medical University, Beijing 100050, China; 5 Chinese Institutes for Medical Research (CIMR), Beijing 100069, China

**Keywords:** ECM remodeling, liver regeneration, matrix–cell signaling

## Abstract

The liver possesses a remarkable regenerative capacity following injury, a process fundamentally orchestrated by the dynamic extracellular matrix (ECM). Far beyond a passive scaffold, the liver matrisome functions as an integrative mechano-biochemical circuit. It comprises a core structural network together with regulatory non-core components that collectively establish a dynamic niche. This niche stores and releases mitogenic cues, transmits mechanical forces, and coordinates multicellular crosstalk. Through receptors like integrins and mechanosensitive channels, ECM-derived signals converge on key pathways, including Hippo-YAP/TAZ and Wnt/β-catenin, to drive hepatocyte proliferation and tissue restructuring. The balance between matrix stabilization and remodeling dictates the outcome, guiding physiological regeneration versus fibrotic progression. Consequently, the ECM emerges as a central therapeutic target and a blueprint for engineering strategies aimed at restoring liver function. Strategies to recalibrate its composition, mechanics, and remodeling, from pharmacological inhibitors to bioengineered decellularized ECM scaffolds, hold significant potential for steering liver repair and combating chronic liver disease.

## 1. Introduction

The liver plays essential roles in systemic metabolism, detoxification and immune homeostasis. In response to acute injury or partial tissue loss, it displays a remarkable capacity for regeneration [1]. Central to this process is the proliferative expansion of hepatocytes, the primary parenchymal cells responsible for restoring liver mass and function [2]. Following partial hepatectomy or acute toxic insult, quiescent hepatocytes rapidly re-enter the cell cycle to replace lost tissue. Concurrently, non-parenchymal cells (NPCs), including sinusoidal endothelial cells (SECs), Kupffer cells (KCs), hepatic stellate cells (HSCs), and biliary epithelial cells (BECs), initiate coordinated angiogenic, inflammatory, and matrix remodeling programs that collectively support hepatocyte proliferation and tissue reorganization [3,4,5]. This highly orchestrated response is governed by temporally regulated cytokine and growth factor networks, together with tightly regulated paracrine communication among liver cell populations [6].

Among the diverse regulatory cues governing liver repair, the extracellular matrix (ECM) plays an indispensable role [5]. Rather than serving as a passive scaffold, the liver ECM represents a dynamic structural and biochemical environment that actively shapes hepatocyte behavior during regeneration [7,8]. Following liver injury, ECM remodeling critically influences the balance between tissue restoration and pathological repair by regulating cell proliferation, migration, and tissue organization [7,9,10]. Appropriate ECM remodeling facilitates hepatocyte regeneration and architectural recovery, whereas excessive or disordered matrix accumulation disrupts these processes and promotes fibrotic progression [9,11]. Thus, the ECM serves not only as a structural framework but also as a central regulator of liver regeneration, ultimately determining whether injury leads to effective repair or chronic fibrosis.

Importantly, the regulatory influence of the ECM arises from its intrinsic plasticity. Changes in matrix composition, physical properties, and remodeling dynamics not only reflect tissue injury but also actively encode instructive signals that can be sensed and integrated by resident liver cells, and are amenable to reprogramming. This tunable nature positions the ECM at the intersection of mechanism and intervention: understanding how ECM-derived biochemical and mechanical cues are generated, perceived, and coordinated provides a conceptual basis for rationally modulating regenerative outcomes. In this review, we synthesize current evidence by examining the ECM through a hierarchical and mechanistic lens. We first discuss how distinct ECM components establish the molecular foundation of the regenerative microenvironment; we then address how ECM mechanics are sensed through mechanotransduction pathways and translated into intracellular signaling programs; finally, we integrate these insights at the tissue level to illustrate how multicellular crosstalk converges on the ECM. This convergence enables the ECM to function as a dynamic decision-making platform that coordinates liver regeneration and fibrotic trajectories.

## 2. Dynamic Roles of the Core and Non-Core Liver Matrisomal Components in Balancing Liver Regeneration and Fibrosis

**Figure 1:**
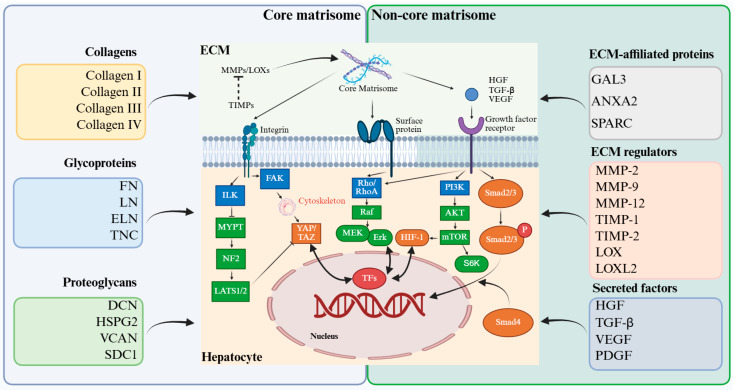
The liver matrisome as an integrative regulator of liver regeneration.

The liver ECM orchestrates regeneration through a hierarchical partnership between its core and non-core matrisome components. At the molecular level, the core matrisome establishes the fundamental structural and biochemical framework of the hepatic microenvironment, while the non-core matrisome refines this framework through enzymatic remodeling, matricellular protein activity, and the spatiotemporal regulation of signaling molecules. Together, these components encode context-dependent biochemical and physical information that shapes cellular responses to injury. The balance between matrix stability and plasticity established at this compositional level critically influences whether liver repair proceeds toward adaptive regeneration or maladaptive fibrotic remodeling (Figure 1).

### 2.1. Core Matrisome

The liver core matrisome, which consists of collagens, glycoproteins, and proteoglycans, forms the fundamental structural and biochemical foundation for liver regeneration [8,12,13]. During the early regenerative phase, activated HSCs and periportal hepatocytes rapidly synthesize fibrillar collagens, such as types I and III, to assemble a provisional scaffold that preserves parenchymal architecture and transmits mechanosensory cues through integrin focal adhesion and cytoskeletal signaling pathways [13,14,15]. This transient collagenous framework not only provides tensile support but also acts as a reservoir for growth factors including hepatocyte growth factor (HGF) and epidermal growth factor (EGF), enabling spatially controlled release that promotes synchronized hepatocyte proliferation [16,17,18]. Concurrently, ECM glycoproteins, most prominently fibronectin, laminins and tenascin C, coordinate cell adhesion, migration, and survival [19,20]. Fibronectin engages integrin α5β1 to activate cytoprotective signaling, whereas laminins and elastin help restore lobular polarity and mechanical elasticity, forming fibrillar conduits that guide the movement of hepatocytes and progenitor cells [21]. Proteoglycans such as decorin (DCN), perlecan and versican (VCAN) further refine the regenerative niche by temporally sequestering and releasing mitogenic factors and buffering profibrotic mediators, including transforming growth factor-β (TGF-β) [22,23].

Collectively, these core ECM components form a dynamic matrix that integrates biochemical gradients with mechanical cues to sustain tissue plasticity and functional recovery [18,24]. The balance between structural stability and matrix remodeling is a defining feature of successful regeneration, distinguishing physiological repair from maladaptive fibrotic responses [25]. The central role of the core matrisome in controlling this transition highlights that it functions not as a passive scaffold but as an active regulator of liver regeneration, guiding the restoration of liver structure and function.

### 2.2. Non-Core Matrisome

Beyond the core matrisome, the non-core matrisome encompasses a broad spectrum of ECM regulators, ECM-affiliated proteins and matrix-bound secreted factors that collectively fine-tune the regenerative niche [26]. Matrix metalloproteinases (MMPs) and their tissue inhibitors (TIMPs) form a tightly regulated proteolytic system that controls the rate of ECM turnover, releasing sequestered mitogens while preventing excessive degradation and structural collapse [27,28]. Complementing this process, lysyl oxidase family enzymes (LOX and LOXL isoforms) catalyze collagen cross-linking to enhance mechanical stability while preserving the pliability required for cell migration and tissue reorganization [29,30,31]. ECM-affiliated proteins such as galectin-3 (GAL3), annexin A2 (ANXA2), and secreted protein acidic and rich in cysteine (SPARC) function as molecular modulators of cell–matrix adhesion, immune-cell recruitment and angiogenic remodeling, thereby linking biochemical cues to tissue morphogenesis [32,33,34]. Simultaneously, the ECM serves as a dynamic reservoir for secreted growth factors such as HGF, TGF-β, vascular endothelial growth factor (VEGF) and platelet-derived growth factor (PDGF), with their bioavailability regulated by proteolytic activity and mechanical inputs [26,35]. The spatially and temporally controlled release of these ligands provides the precision necessary for synchronized hepatocyte proliferation and vascular repair [36].

Collectively, these non-core matrisome components transform the ECM into an information-rich signaling interface that integrates proteolytic remodeling, mechanical feedback, and paracrine crosstalk [37]. Through these mechanisms, they critically influence the balance between adaptive regeneration and pathological scar formation. However, the regulatory capacity of these components is not solely determined by their presence or abundance. The same molecular constituents can exert distinct effects depending on their organization, cross-linking, and mechanical context, thereby raising the question of how liver cells sense and interpret the physical state of the ECM during regeneration. Addressing this question requires consideration of ECM mechanics and mechanotransduction.

## 3. Liver ECM Orchestrates Regeneration Through a Mechano-Biochemical Circuit

The ECM that emerges after liver injury is not a passive scaffold, but an instructive niche that translates compositional changes into mechanical signals guiding tissue regeneration [24,38,39]. At this level, ECM mechanics function as a critical interface through which biochemical information encoded in matrix composition is sensed and interpreted by resident liver cells. Activation of LOX family enzymes (LOX and LOXL1–4) promotes covalent cross-linking of type I and type III collagens, strengthening the newly formed matrix and modulating its mechanical properties [40,41]. This stabilization preserves the cohesion of the regenerating parenchyma while avoiding excessive stiffening that would favor fibrotic progression [42,43]. In parallel, MMPs such as MMP-2 and MMP-9, along with related proteases, remodel the ECM by cleaving overly stabilized fibrils and releasing matrix-bound morphogens, including HGF, VEGF, and epidermal growth factor (EGF) [12,42]. The dynamic balance between LOX-mediated stabilization and MMP-driven remodeling defines an optimal microenvironment that harmonizes stiffness, porosity, and ligand availability to support hepatocyte adhesion, polarization, and coordinated proliferation [41,44].

Hepatocytes and NPCs sense these physical cues through specialized mechanosensors. Integrins (e.g., α5β1, αvβ3) clustered in focal adhesions recruit focal adhesion kinase (FAK) and Proto-oncogene tyrosine-protein kinase Src, activating the RAS-RAF-MEK-ERK and PI3K-AKT-mTOR cascades to promote anabolic growth and survival [40,45]. Concurrently, integrin engagement stimulates RhoA–ROCK–mediated actomyosin contractility, amplifying FAK signaling in a positive feedback loop that refines focal adhesion maturation and strength [46]. Mechanical shear stress and tensile strain are also sensed by mechanosensitive ion channels such as Piezo1 and TRPV4; subsequent Ca^2+^ influx activates calcineurin-NFAT and CaMKII/PKC-MAPK pathways, reinforcing proliferation-associated transcription and promoting Yes-associated protein/transcriptional coactivator with PDZ-binding motif (YAP/TAZ) dephosphorylation [47,48]. Central to this network is the Hippo-YAP/TAZ axis: increased cytoskeletal tension or matrix rigidity suppresses MST1/2-LATS1/2 kinase activity, enabling YAP and TAZ to translocate into the nucleus; there, they partner with TEA domain transcription factor family (TEAD) transcription factors to drive the expression of pro-proliferative genes such as *Cyclin D1*, connective tissue growth factor (*CTGF*), cysteine-rich angiogenic inducer 61 (*Cyr61*), and amphiregulin (*AREG*) [46,49]. This mechanical circuitry further converges with canonical Wnt/β-catenin signaling, disrupting the destruction complex (Axin/GSK3β) and stabilizing β-catenin, which then cooperates with T-cell factor (TCF), and is further potentiated by YAP/TAZ activation, to promote transcriptional synergy that drives hepatocytes through the G1/S transition and restores liver mass [40,44].

Mechanical homeostasis is equally crucial during the resolution phase. Persistent LOX activity or excessive cross-linking locks the ECM into a rigid, fibrotic state, restricting hepatocyte migration and sustaining HSC activation [43]. Conversely, broad MMP inhibition compromises matrix clearance and growth factor release [12]. Disruption of integrin-FAK signaling or enforced Hippo pathway activation impairs YAP/TAZ-dependent transcription and delays tissue recovery [34]. Restoring optimal matrix compliance, achieved through balanced LOX/MMP activity and tuned actomyosin tension, reactivates FAK-ERK/AKT, YAP/TAZ-TEAD, and β-catenin-TCF programs in a coordinated manner [34,43]. Together, these findings support a unified framework in which the liver ECM functions as an integrated mechano-biochemical circuit, synchronizing matrix stiffness, calcium flux, and transcriptional crosstalk to orchestrate regeneration [50]. The liver exemplifies, perhaps more clearly than any other organ, that successful repair depends not only on biochemistry but also on biomechanics [51,52].

**Figure 2:**
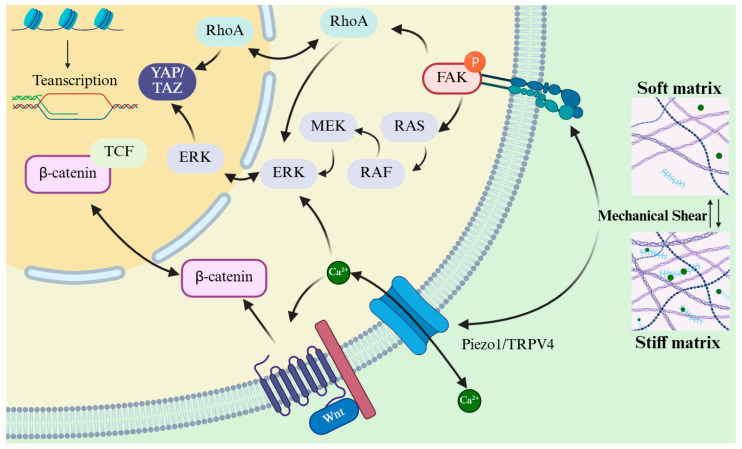
Mechanical signaling networks in liver regeneration.

Taken together, the liver’s regenerative capability is fundamentally guided by a mechano-biochemical network centered on the dynamic ECM (Figure 2). This system establishes a tunable mechanical microenvironment through balanced LOX- and MMP-mediated remodeling, creating an optimal physical niche for hepatocyte proliferation. Mechanical cues are transduced via integrin-mediated pathways and mechanosensitive ion channels, converging on key transcriptional regulators including YAP/TAZ, β-catenin, and ERK-dependent signaling to coordinate proliferative and morphogenetic programs. This regulatory network enables transient physical stimuli to elicit sustained pro-regenerative responses, yet the same mechanisms can promote fibrosis and pathogenesis under conditions of sustained mechanical imbalance. Future therapeutic strategies aimed at restoring regenerative capacity will need to target this intricate mechanochemical code that bridges physical cues with biological outcomes in liver repair. Importantly, these mechanotransduction pathways operate within a multicellular context. Hepatocytes, stromal cells, endothelial cells, and immune populations simultaneously sense, modify, and respond to ECM mechanics, necessitating coordinated signal integration at the tissue level. Understanding how ECM-derived mechanical information is integrated across distinct cell types is therefore essential for explaining how regenerative programs are initiated, amplified, and ultimately terminated during liver repair.

## 4. Liver ECM Coordinates Liver Regeneration Through Multicellular Crosstalk

**Figure 3:**
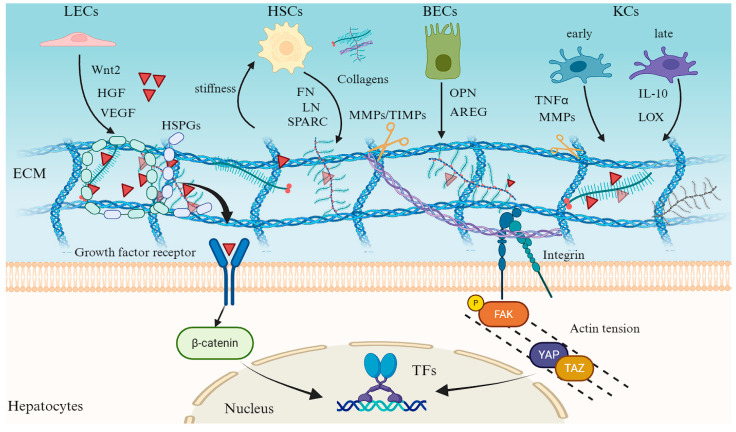
Multicellular crosstalk mediated by the liver ECM during regeneration.

Following acute liver injury or partial hepatectomy, the liver ECM rapidly transitions from a source of cell-specific mechanical cues into a shared tissue-level signaling hub. This reprogrammed microenvironment integrates biochemical, mechanical, and immunological cues to direct multicellular regeneration [51,52]. Its transient remodeling is sustained through intricate crosstalk among hepatocytes, NPCs, and BECs, each contributing distinct ECM-modifying and -sensing capabilities [53,54] (Figure 3).

Liver SECs act as early orchestrators of the regenerative niche. Through the release of angiocrine factors, including Wnt2, HGF, and VEGF, they stimulate hepatocyte proliferation and remodel the surrounding matrix through pathways involving urokinase-type plasminogen activator (uPA) and MMPs [55,56,57,58]. These secreted factors are sequestered within the ECM by heparan sulfate proteoglycans and are subsequently released in response to local mechanical and proteolytic signals, creating spatially resolved mitogenic gradients that align with sinusoidal flow [59,60].

Concurrently, HSCs sense alterations in ECM stiffness and composition through integrin-FAK and YAP/TAZ-mediated mechanotransduction [61,62]. This prompts their transition into a transiently activated, pro-regenerative phenotype. In this state, HSCs deposit fibronectin, laminin, and matricellular proteins such as SPARC and osteopontin (OPN), which provide both adhesive ligands and mechanical elasticity to facilitate hepatocyte spreading, ductular cell migration, and vascular stabilization [63,64,65,66]. These HSC-derived matrices also act as molecular scaffolds that coordinate growth-factor presentation and regulate endothelial permeability, thereby linking mechanical feedback to metabolic and transcriptional reprogramming in regenerating hepatocytes [67,68].

BECs, traditionally regarded as passive ductal components, are now recognized as pivotal contributors to ECM remodeling and regenerative patterning. Upon injury, BECs upregulate the secretion of matricrine mediators such as AREG, OPN, and CTGF, which bind ECM glycosaminoglycans and enhance hepatocyte proliferation at the ductal-parenchymal interface [69,70,71,72]. Simultaneously, BECs release MMPs and TIMPs, enabling finely tuned ECM turnover that facilitates ductular-reaction expansion and the migration of progenitor-like cells into periportal zones [73,74,75]. Crosstalk between BECs and KCs further amplifies this process: KC-derived Wnt ligands sustain BECs-to-hepatocytes differentiation, whereas BEC-derived cytokines such as IL-6 and OPN recruit KCs and shape their activation state toward a reparative phenotype [6,76,77].

KCs and infiltrating monocyte-derived macrophages serve as temporal regulators of ECM homeostasis. During the early inflammatory phase, these macrophages release tumor necrosis factor-α (TNF-α), MMP-9, and MMP-12 to degrade necrotic ECM and release matrix-bound mitogens such as HGF and VEGF [78,79,80]. As regeneration proceeds, macrophages shift to an IL-10–dominant, M2-like state, promoting ECM reassembly through induction of collagen cross-linking enzymes (LOX family) and secretion of anti-fibrotic mediators [81,82,83]. This biphasic pattern of ECM degradation and reconstitution ensures that hepatocytes and BECs are exposed to temporally ordered mechanical and biochemical cues—initially permissive for proliferation, subsequently restrictive to facilitate termination and remodeling [84,85,86].

Ultimately, these intercellular interactions transform the ECM into a self-regulating mechano-biochemical network. Through integrin signaling, cytoskeletal tension, and mechanosensitive transcriptional programs such as YAP/TAZ and β-catenin, hepatocytes and NPCs continuously interpret and reshape their matrix context. The resulting regenerative circuit integrates endothelial angiocrine signaling, HSC mechanotransduction, macrophage-driven remodeling, and cholangiocyte plasticity to restore liver architecture and function with remarkable precision. Viewed in this light, liver regeneration exemplifies a paradigm in which ECM dynamics function as both the language and the logic of multicellular coordination, translating injury-induced physical and molecular perturbations into an ordered program of tissue reconstruction. Importantly, the adaptive nature of this ECM-centered network suggests that regenerative outcomes are not fixed but potentially tunable, providing a conceptual foundation for strategies aimed at modulating the matrix microenvironment to favor repair over fibrosis.

## 5. ECM as a Therapeutic Target and Engineering Blueprint Orchestrating Liver Regeneration

In light of the molecular, mechanical, and cellular processes described above, the ECM has increasingly been considered a relevant entry point for therapeutic modulation in liver regeneration [87,88]. Accordingly, a range of intervention strategies has been explored to influence liver repair by adjusting ECM composition, cross-linking, or mechanical properties following injury or resection [89]. These ECM-directed approaches include molecular modulation of matrix remodeling and mechanotransduction pathways, as well as bioengineering platforms designed to recapitulate or modify selected features of the native liver ECM (Table 1).

Pharmacologic targeting of fibrogenic signaling constitutes a major class of ECM-directed therapies. Within this framework, selected interventions targeting the TGF-β axis exert direct pro-regenerative effects in addition to antifibrotic activity. As a master regulator of HSC activation and SMAD2/3-driven matrix deposition, TGF-β signaling is antagonized by recombinant decorin, which sequesters extracellular ligands, and by LY2157299, a selective TGF-β receptor I inhibitor, both of which are associated with enhanced hepatocyte proliferation and accelerated liver mass recovery following partial hepatectomy or toxic injury [90,91,92]. Other modulators of this pathway, including SB525334, phosphocreatine, 3-HBI, and astaxanthin, primarily attenuate fibrogenic and oxidative injury, thereby relieving microenvironmental constraints that permit, rather than directly induce, endogenous regeneration [93,94,95,96]. Therapeutic targeting of ECM biomechanics further modulates regenerative competence. Collagen cross-linking mediated by LOX and LOXL enzymes drives matrix stiffening and limits tissue compliance, thereby constraining hepatocyte renewal [29]. Selective inhibition of LOXL2 using AB0023 or GS341 directly couples matrix softening with hepatocyte proliferation, whereas broader LOX inhibition predominantly suppresses pathological remodeling and injury, exerting indirect pro-regenerative effects [31,97,98,99]. Dual LOXL2/LOXL3 inhibition with PXS-5153A similarly improves matrix compliance, establishing a biomechanical milieu permissive for regeneration [100]. Dynamic regulation of ECM turnover constitutes an additional axis of regenerative control. Augmentation of matrix degradation through *MMP-13* gene delivery or MMP-9 suppression promotes hepatocyte cell-cycle re-entry and liver regeneration, in part by releasing matrix-sequestered mitogenic cues and restoring ECM plasticity [101,102,103]. Finally, canonical regenerative signaling pathways are tightly interwoven with ECM remodeling. Normalization of aberrant Wnt activity using DKK1 or sFRP5 restores hepatocyte proliferative capacity, while disruption of β-catenin-CBP-dependent transcription with ICG-001 robustly enhances hepatocyte proliferation across experimental systems [104,105,106]. PRI-724 demonstrates early clinical activity in HCV-associated fibrosis, consistent with enhanced regenerative competence despite limited direct proliferation readouts [107,108]. Recombinant BMP-7 represents a bona fide pro-regenerative cue following partial hepatectomy, acting through stromal reprogramming and coordinated ECM remodeling to support hepatocyte renewal [109].

**Table 1:** Summary of ECM-targeted strategies for liver regeneration.

Category	Target	Intervention	Models	Effect	References
Molecular targeting of ECM dynamics	TGF-β	Recombinant decorin (binds to TGF-β)	PHx or CCl_4_ mouse models	++	[90]
		LY2157299	PHx or CCl_4_ mouse models	+	[91,92]
	LOXl2	AB0023	TAA or DDC mouse models	+++	[31]
		GS341	CCl_4_ mouse models	++	[98]
	MMP-13 MMP-9	MMP13-encoding plasmids (pBGI-MMP13)	TAA mouse models	++	[101]
		MMP-9 antisense oligonucleotides	PHx rat models	++	[103]
	Wnt3a/4/5a Wnt5a	DKK1	BDL mouse models, primary mouse HSCs	++	[104]
		sFRP5	CCl_4_ mouse models	+	[105]
	Wnt pathway	ICG-001	CCl_4_ mouse models, LX-2 cells, primary human fibroblasts	++	[106]
	Wnt	PRI-724	Human HCV	+	[108]
	BMP-7	Recombinant human BMP-7 (rhBMP-7)	PHx mouse models	+	[109]
ECM-based tissue engineering strategies	dECM	Injectable hydrogel	Rat models of liver ischemia/reperfusion injury	++	[110]
		L-ECM hydrogel	TAA rat models	++	[110]
		dECM-Cryogel implantable scaffold	Liver failure rat models	+	[111]
		Self-assembling dECM hydrogel adhesive	Rabbit models of hemorrhage liver injury	++	[112]
		dECM-based bioengineered lobules	Mouse models of acute liver failure	++	[113]
		THBS1-dECM	PHx mouse models, primary mouse hepatocytes	+++	[114]
		L-ECM hydrogel	Rat primary hepatocytes	++	[115]

The observed effect was categorized into three levels based on the percentage of response: + (0–25%), ++ (26–50%), +++ (51–75%). Outcomes reflect relative improvement in liver regeneration parameters (e.g., hepatocyte proliferation, liver mass recovery, or fibrosis resolution) as reported in the original studies.

In parallel, bioengineering strategies increasingly seek to reconstruct the instructive roles of liver ECM. Among these, decellularized ECM (dECM) hydrogels and scaffolds are the most established platforms, retaining organ-specific biochemical complexity and supporting hepatocyte adhesion, vascularization, and ductal morphogenesis in preclinical models [26,110,111]. Advanced dECM configurations, such as cryogelated scaffolds and nanoparticle-functionalized ECM hydrogels, restore liver architecture while modulating local immune responses [112]. Injectable immunoregulatory hydrogels promote hepatoprotective macrophage phenotypes in ischemia–reperfusion models, whereas THBS1-enriched dECM bioinks enhance liver organoid maturation by recapitulating developmental cues [113,116]. Other formulations, including self-assembling dECM adhesives, bioengineered lobular constructs, and hydrogels optimized for hepatocyte transplantation or culture, further expand the regenerative toolkit [114,115,117,118]. As dECM platforms evolve in biological specificity and design flexibility, they are increasingly viewed not as passive scaffolds, but as programmable matrices capable of directing liver repair.

Collectively, these ECM-centered molecular and engineering innovations converge on a common principle: effective liver regeneration depends on the restoration of a compliant, growth-factor-responsive, and dynamically remodeled ECM. By simultaneously recalibrating biochemical signaling and mechanical feedback, ECM-targeted therapeutics and biomimetic scaffolds hold transformative potential for clinical translation, offering regenerative platforms that not only repair but actively reprogram the injured liver toward physiological homeostasis.

## 6. Discussion

Despite extensive evidence supporting a regulatory role for the ECM in liver regeneration, the functional consequences of ECM remodeling are highly context dependent and, in some cases, appear contradictory [111]. Transient increases in matrix stiffness during early regenerative phases are associated with enhanced hepatocyte proliferation and tissue reorganization, whereas sustained or excessive stiffening consistently correlates with fibrotic progression and impaired repair [44,112]. A similar duality characterizes ECM-regulated mechanotransduction pathways, including YAP/TAZ and β-catenin signaling, which can support regenerative responses when activated in a temporally controlled manner but contribute to ductular reactions, fibrosis, or tumorigenic risk when persistently engaged [48,62,113]. Matrix remodeling enzymes further illustrate this balance: LOX-mediated cross-linking is required to preserve tissue integrity during regeneration, yet excessive cross-linking promotes mechanical locking, while MMP-driven proteolysis facilitates remodeling and growth factor release but may disrupt tissue architecture if unrestrained [30,44,81,103]. Collectively, these observations indicate that ECM-mediated regulation of liver regeneration operates within dynamic thresholds rather than uniform pro- or anti-regenerative programs.

Many apparent inconsistencies in the literature can be attributed to methodological and experimental constraints that limit cross-study interpretation. Liver injury models differ substantially in inflammatory burden, spatial patterns of ECM remodeling, and regenerative kinetics, complicating direct comparison of ECM-associated effects across experimental systems [24,116,117]. Moreover, ECM properties are frequently assessed using bulk measurements that obscure spatial heterogeneity within the regenerating liver, despite mounting evidence that localized differences in stiffness, composition, and remodeling dynamics exert cell-type–specific effects [111,114,115]. In vitro platforms similarly struggle to recapitulate the reciprocal and evolving interactions between cells and matrix observed in vivo, as static substrates with fixed stiffness fail to capture the dynamic mechanical landscape characteristic of regeneration [118]. In addition, most ECM-targeted interventions lack cellular specificity, making it difficult to disentangle direct effects on hepatocytes from indirect contributions of stromal, endothelial, or immune populations. Together, these limitations constrain the causal interpretation of ECM–cell interactions and underscore the need for higher-resolution and context-aware experimental approaches.

In light of these considerations, focusing on individual ECM components or signaling pathways in isolation may be insufficient to explain regenerative outcomes. An emerging alternative is to conceptualize the ECM in terms of dynamic states defined by coordinated changes in composition, mechanics, and remodeling activity. Within this framework, permissive ECM states support hepatocyte proliferation, angiogenesis, and tissue reorganization, whereas maladaptive states favor mechanical confinement, chronic inflammation, and fibrotic progression. Advancing this perspective will require integrative strategies that combine spatially resolved transcriptomic and proteomic analyses with quantitative measurements of matrix mechanics, alongside programmable biomaterials capable of systematically interrogating defined ECM conditions [119]. By shifting emphasis from single molecular targets to ECM states, this framework offers a coherent means to reconcile divergent findings across models and experimental contexts.

Despite the mechanistic insights summarized above, several key knowledge gaps remain. The temporal boundaries that distinguish permissive from maladaptive ECM states are poorly defined, particularly across different injury etiologies and phases of regeneration. How distinct liver cell populations differentially sense, remodel, and respond to evolving ECM states in vivo also remains incompletely understood, owing in part to limitations in cell-type–specific and spatially resolved analyses. In addition, current approaches for measuring ECM mechanics and composition often lack the resolution required to capture local heterogeneity and dynamic remodeling, constraining causal inference. Finally, the long-term consequences of experimentally manipulating ECM properties—including tissue stability, reversibility, and potential oncogenic risk—have not been systematically evaluated. Addressing these gaps will require coordinated application of spatial omics, quantitative biomechanics, and dynamic in vitro and in vivo models capable of capturing ECM–cell reciprocity over regenerative time courses.

## 7. Conclusions

In conclusion, liver regeneration highlights the remarkable capacity of adult tissues to restore structure and function through the coordinated remodeling of the ECM. As discussed in this review, ECM composition, mechanics, and multicellular interactions jointly shape regenerative trajectories in a context- and time-dependent manner, with distinct matrix states supporting either effective repair or fibrotic progression. A central challenge moving forward is to move beyond reductionist descriptions of individual ECM components and instead define how biochemical cues, mechanical properties, and intercellular signaling are integrated within the matrix microenvironment. Advances in spatial omics, quantitative biomechanics, and engineered model systems are expected to enable more precise characterization of these ECM states. Translationally, while ECM-directed molecular interventions and biomaterial-based strategies show promise in modulating regenerative microenvironments, substantial work remains to establish their stability, specificity, and long-term safety. By framing the ECM as a tunable but tightly constrained regulatory system, future efforts in regenerative hepatology may better align mechanistic insight with rational therapeutic design.

## Data Availability

No new data were created or analyzed in this study.

## Abbreviations

The following abbreviations are used in this manuscript:

ANXA2	Annexin A2
AREG	Amphiregulin
BDL	Bile duct ligation
BECs	Biliary epithelial cells
BMP-7	Bone morphogenetic protein 7
BAPN	β-aminopropionitrile
CTGF	Connective tissue growth factor
CYR61	Cysteine-rich angiogenic inducer 61
dECM	Decellularized extracellular matrix
DCN	Decorin
DDC	3,5-diethoxycarbonyl-1,4-dihydrocollidine model
ECM	Extracellular matrix
EGF	Epidermal growth factor
EV	Extracellular vesicle
FAK	Focal adhesion kinase
GAL3	Galectin-3
GSK3β	Glycogen synthase kinase 3 beta
HA	Hyaluronic acid
HCV	Hepatitis C virus
HGF	Hepatocyte growth factor
HSC	Hepatic stellate cell
KC	Kupffer cell
LOX/LOXL	Lysyl oxidase/lysyl oxidase-like enzymes
LSEC	Liver sinusoidal endothelial cell
MMPs	Matrix metalloproteinases
NASH	Non-alcoholic steatohepatitis
NFAT	Nuclear factor of activated T cells
OPN	Osteopontin
PDGF	Platelet-derived growth factor
PHx	Partial hepatectomy
RAF	Rapidly accelerated fibrosarcoma kinase
RAS	Rat sarcoma family GTPase
SPARC	Secreted protein acidic and rich in cysteine
SPP1	Secreted phosphoprotein 1
TAA	Thioacetamide
TCF	T-cell factor
TEAD	TEA domain transcription factor family
TIMPs	Tissue inhibitors of metalloproteinases
TRPV4	Transient receptor potential vanilloid 4
VCAN	Versican
VEGF	Vascular endothelial growth factor
